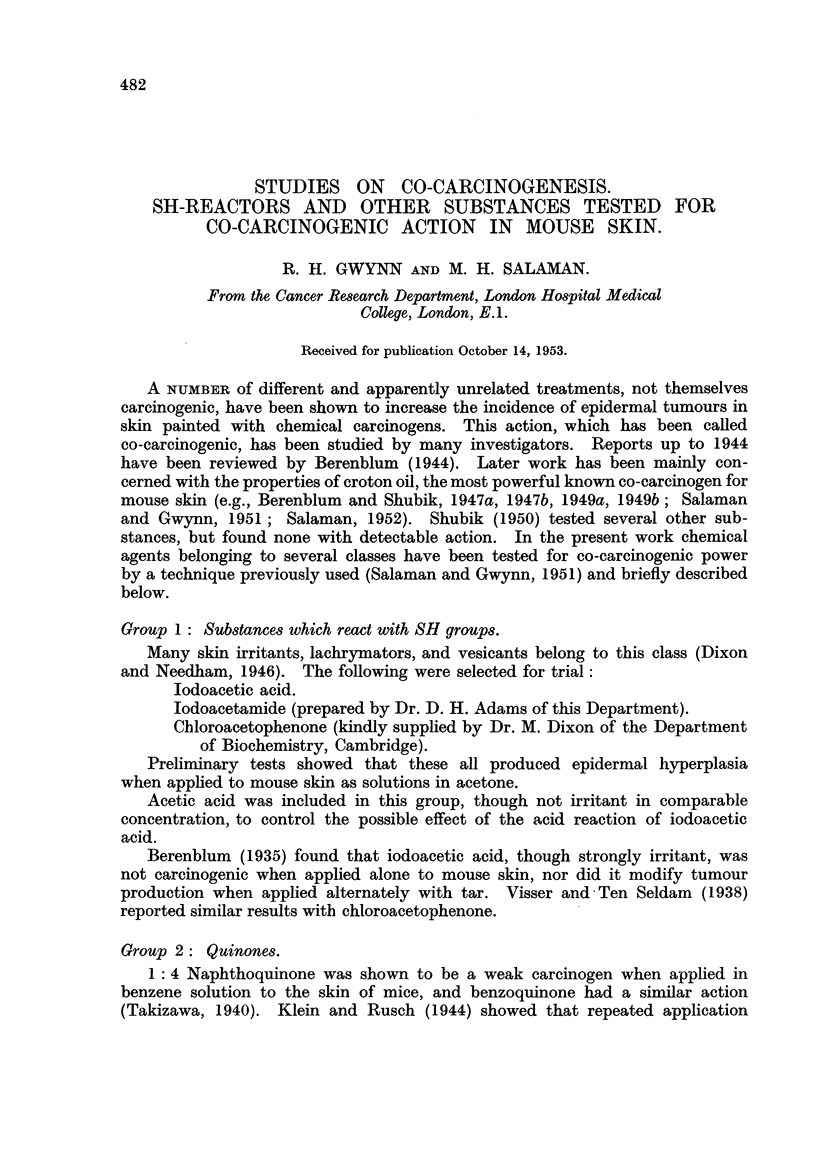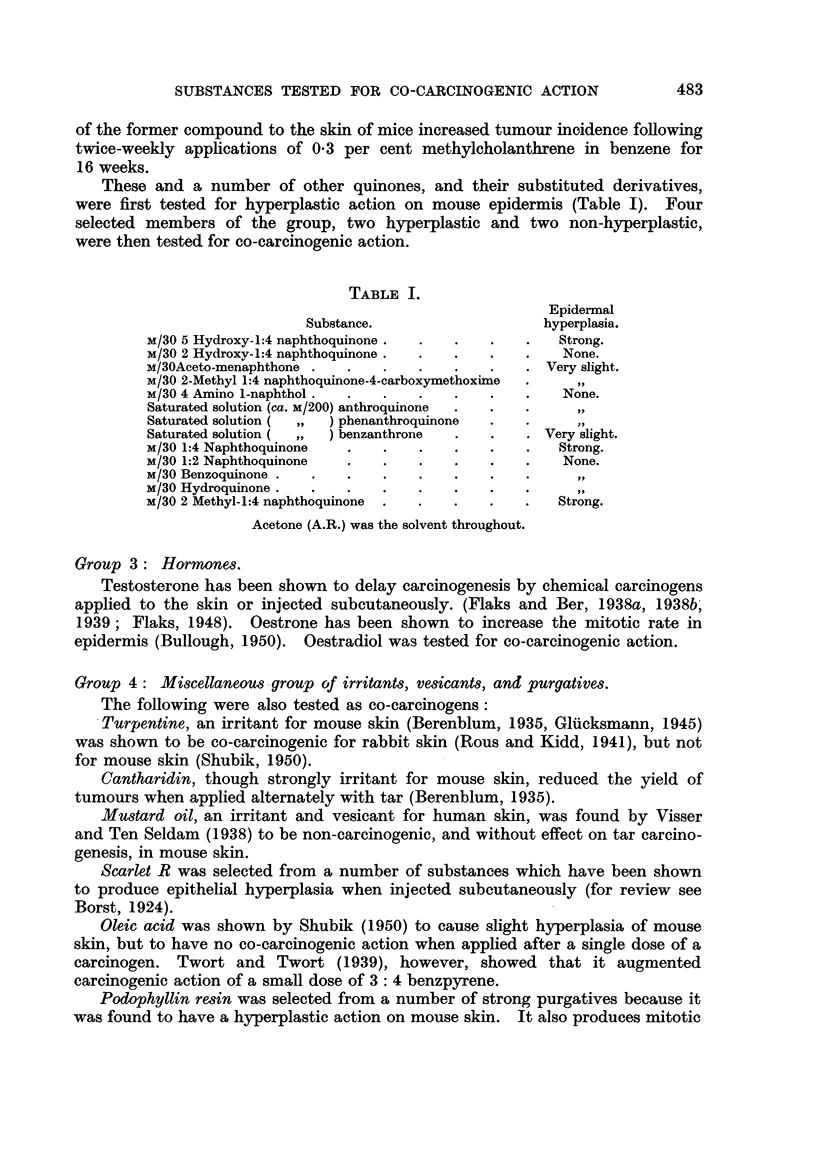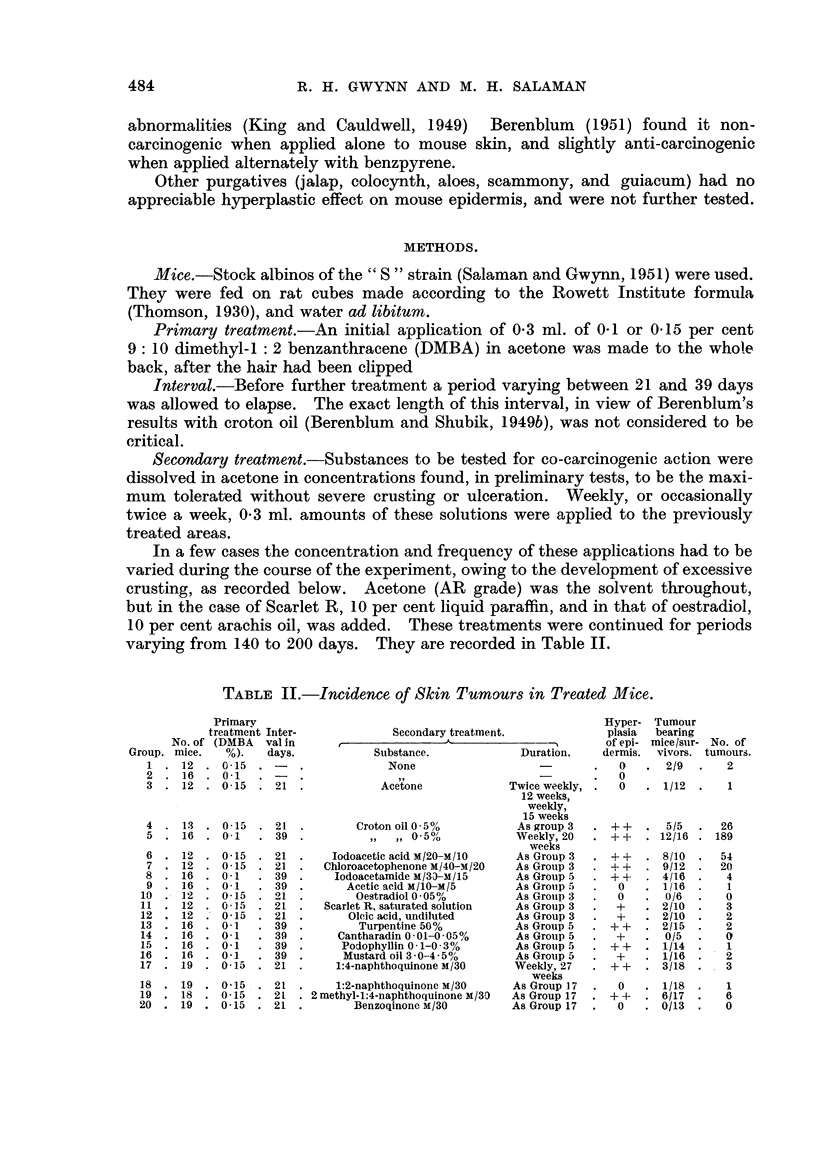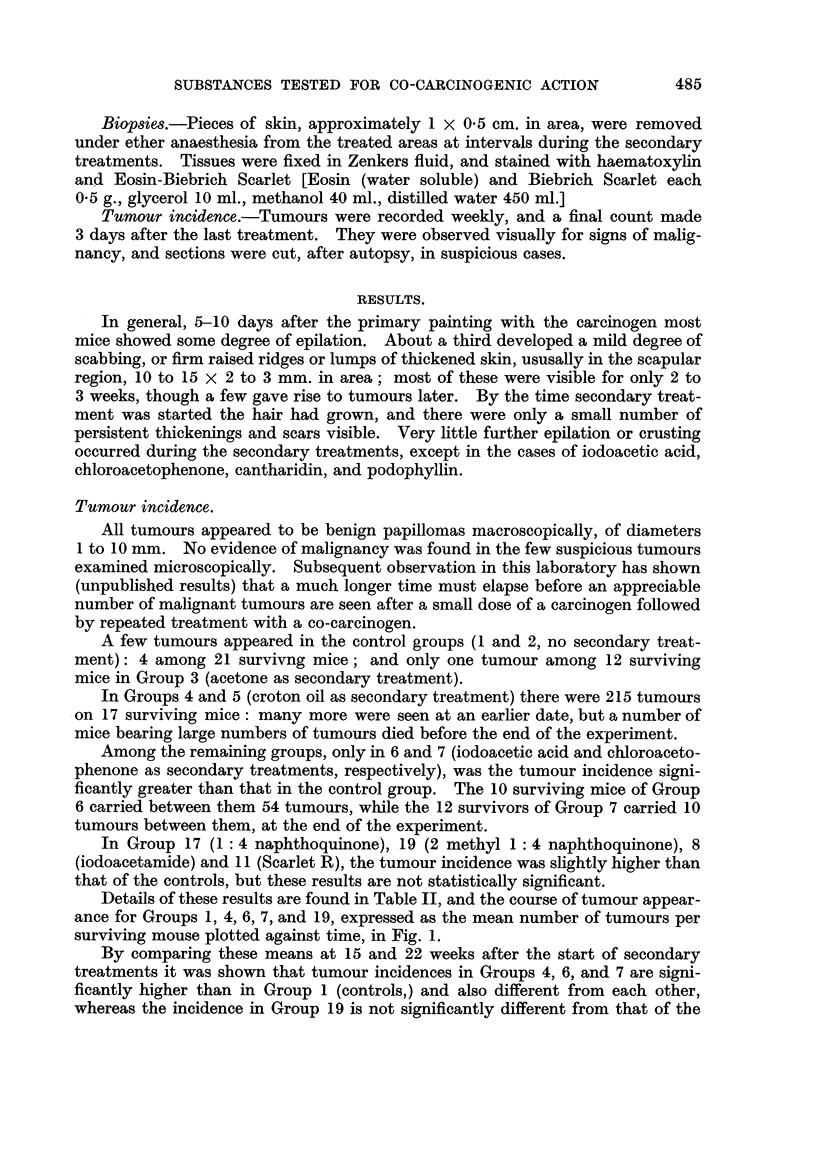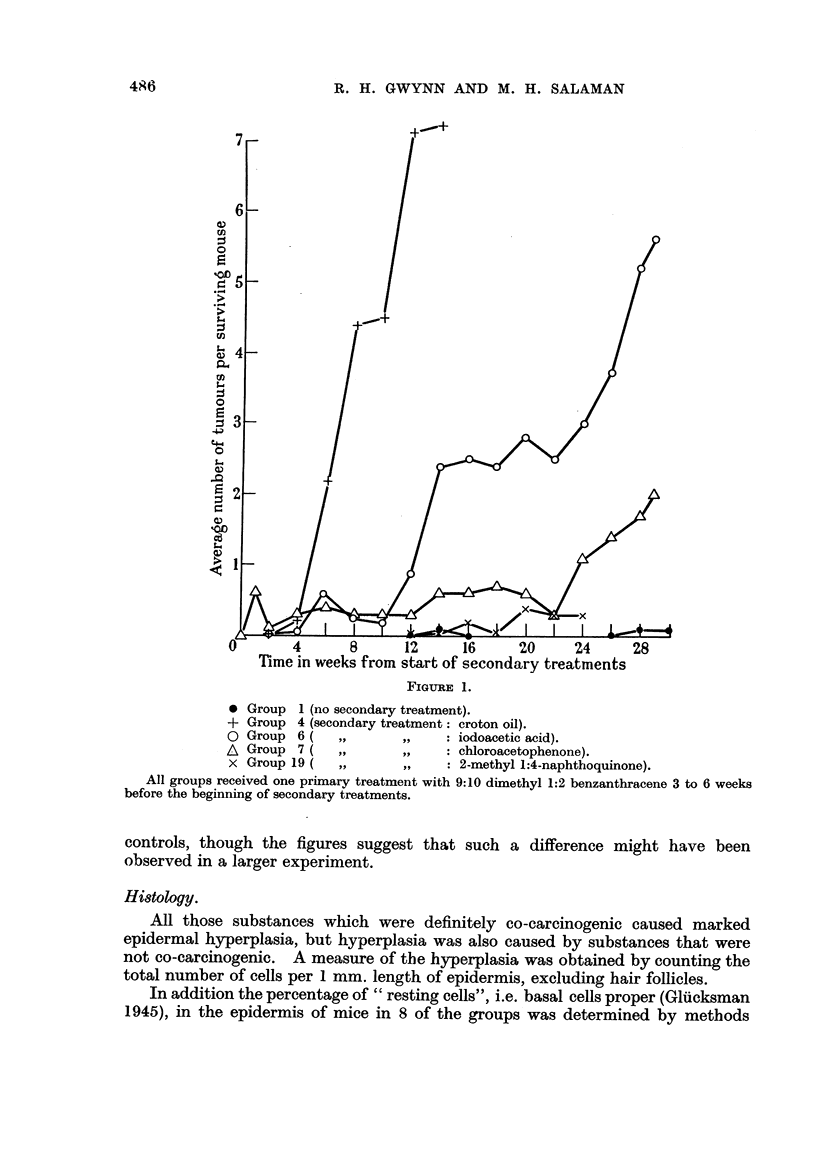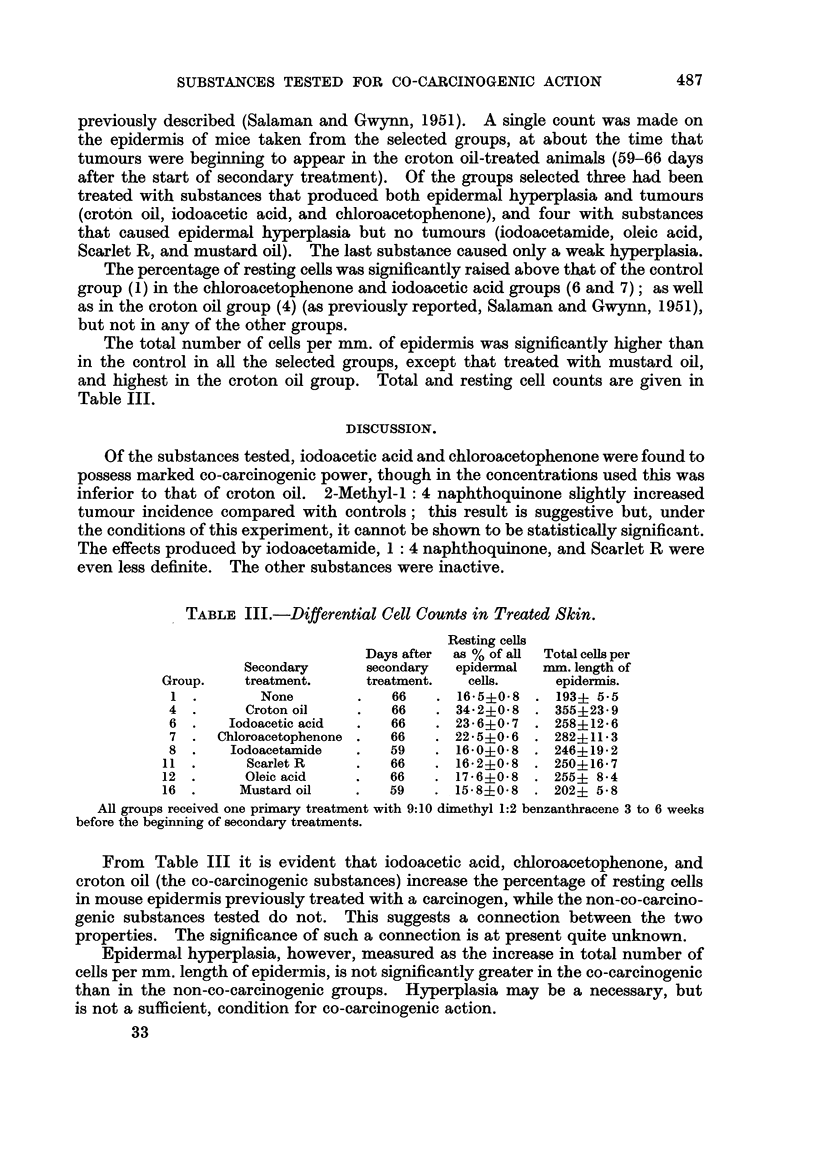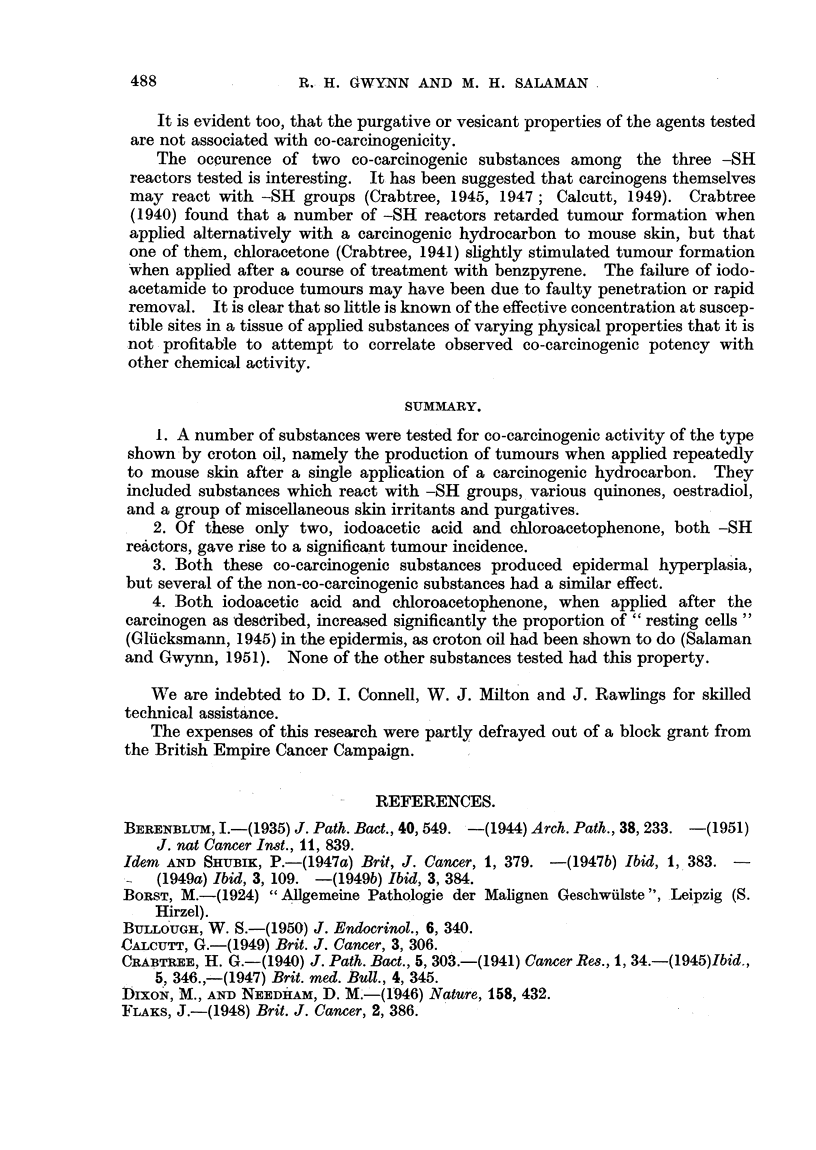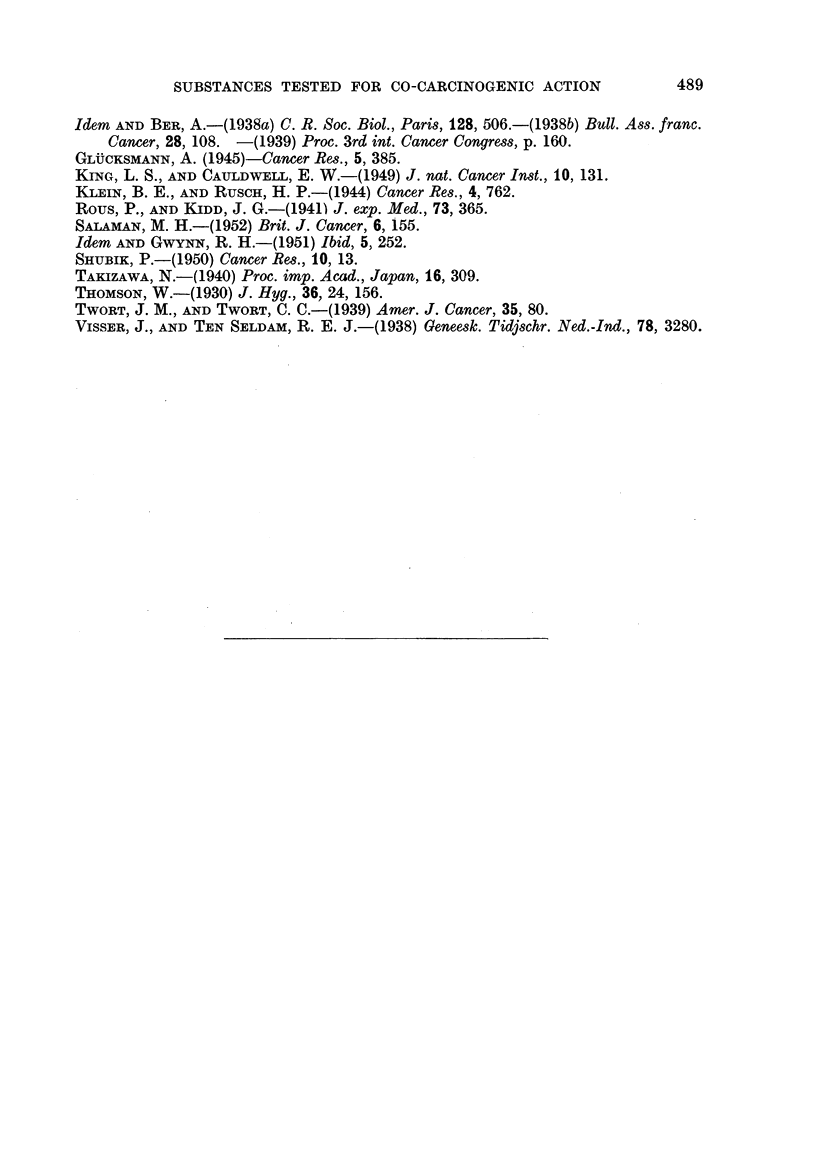# Studies on Co-carcinogenesis. SH-Reactors and other Substances Tested for Co-carcinogenic action in Mouse Skin

**DOI:** 10.1038/bjc.1953.50

**Published:** 1953-12

**Authors:** R. H. Gwynn, M. H. Salaman


### STUDIES ON CO-CARCINOGENESIS. SH-REACTORS AND OTHER SUBSTANCES TESTED FOR CO-CARCINOGENIC ACTION IN MOUSE SKIN.

R. H. GWYNN AND M. H. SALAMAN.

*From the Cancer Research Department, London Hospital Medical College, London, E.*1.

Received for publication October 14, 1953.

A NUMBER of different and apparently unrelated treatments, not themselves carcinogenic, have been shown to increase the incidence of epidermal tumours in skin painted with chemical carcinogens. This action, which has been called co-carcinogenic, has been studied by many investigators. Reports up to 1944 have been reviewed by Berenblum (1944). Later work has been mainly concerned with the properties of croton oil, the most powerful known co-carcinogen for mouse skin (e.g., Berenblum and Shubik, 1947*a*, 1947*b*, 1949*a*, 1949*b* ; Salaman and Gwynn, 1951 ; Salaman, 1952). Shubik (1950) tested several other substances, but found none with detectable action. In the present work chemical agents belonging to several classes have been tested for co-carcinogenic power by a technique previously used (Salaman and Gwynn, 1951) and briefly described below.

*Group* 1 : *Substances which react with SH groups.*

Many skin irritants, lachrymators, and vesicants belong to this class (Dixon and Needham, 1946). The following were selected for trial :

Iodoacetic acid.

Iodoacetamide (prepared by Dr. D. H. Adams of this Department).

Chloroacetophenone (kindly supplied by Dr. M. Dixon of the Department of Biochemistry, Cambridge).

Preliminary tests showed that these all produced epidermal hyperplasia when applied to mouse skin as solutions in acetone.

Acetic acid was included in this group, though not irritant in comparable concentration, to control the possible effect of the acid reaction of iodoacetic acid.

Berenblum (1935) found that iodoacetic acid, though strongly irritant, was not carcinogenic when applied alone to mouse skin, nor did it modify tumour production when applied alternately with tar. Visser and Ten Seldam (1938) reported similar results with chloroacetophenone.

*Group* 2 : *Quinones.*

1 : 4 Naphthoquinone was shown to be a weak carcinogen when applied in benzene solution to the skin of mice, and benzoquinone had a similar action (Takizawa, 1940). Klein and Rusch (1944) showed that repeated application of the former compound to the skin of mice increased tumour incidence following twice-weekly applications of 0·3 per cent methylcholanthrene in benzene for 16 weeks.

These and a number of other quinones, and their substituted derivatives, were first tested for hyperplastic action on mouse epidermis (Table I). Four selected members of the group, two hyperplastic and two non-hyperplastic, were then tested for co-carcinogenic action.

TABLE I.

| Substance. | Epidermal hyperplasia. |
|---|---|
| M/30 5 Hydroxy-1:4 naphthoquinone . . . . . . | Strong. |
| M/30 2 Hydroxy-1:4 naphthoquinone . . . . . . | None. |
| M/30Aceto-menaphthone . . . . . . . . | Very slight. |
| M/30 2-Methyl 1:4 naphthoquinone-4-carboxymethoxime . | ,, |
| M/30 4 Amino 1-naphthol . . . . . . . . | None. |
| Saturated solution (*ca.* M/200) anthroquinone . . . | ,, |
| Saturated solution ( ,, ) phenanthroquinone . . | ,, |
| Saturated solution ( ,, ) benzanthrone . . . | Very slight. |
| M/30 1:4 Naphthoquinone . . . . . . . | Strong. |
| M/30 1:2 Naphthoquinone . . . . . . . | None. |
| M/30 Benzoquinone . . . . . . . . . . | ,, |
| M/30 Hydroquinone . . . . . . . . . . | ,, |
| M/30 2 Methyl-1:4 naphthoquinone . . . . . . | Strong. |

Acetone (A.R.) was the solvent throughout.

*Group* 3 : *Hormones.*

Testosterone has been shown to delay carcinogenesis by chemical carcinogens applied to the skin or injected subcutaneously. (Flaks and Ber, 1938*a*, 1938*b*, 1939 ; Flaks, 1948). Oestrone has been shown to increase the mitotic rate in epidermis (Bullough, 1950). Oestradiol was tested for co-carcinogenic action.

*Group* 4 : *Miscellaneous group of irritants, vesicants, and purgatives.*

The following were also tested as co-carcinogens :

*Turpentine*, an irritant for mouse skin (Berenblum, 1935, Glücksmann, 1945) was shown to be co-carcinogenic for rabbit skin (Rous and Kidd, 1941), but not for mouse skin (Shubik, 1950).

*Cantharidin*, though strongly irritant for mouse skin, reduced the yield of tumours when applied alternately with tar (Berenblum, 1935).

*Mustard oil*, an irritant and vesicant for human skin, was found by Visser and Ten Seldam (1938) to be non-carcinogenic, and without effect on tar carcinogenesis, in mouse skin.

*Scarlet R* was selected from a number of substances which have been shown to produce epithelial hyperplasia when injected subcutaneously (for review see Borst, 1924).

*Oleic acid* was shown by Shubik (1950) to cause slight hyperplasia of mouse skin, but to have no co-carcinogenic action when applied after a single dose of a carcinogen. Twort and Twort (1939), however, showed that it augmented carcinogenic action of a small dose of 3 : 4 benzpyrene.

*Podophyllin resin* was selected from a number of strong purgatives because it was found to have a hyperplastic action on mouse skin. It also produces mitotic abnormalities (King and Cauldwell, 1949) Berenblum (1951) found it non-carcinogenic when applied alone to mouse skin, and slightly anti-carcinogenic when applied alternately with benzpyrene.

Other purgatives (jalap, colocynth, aloes, scammony, and guiacum) had no appreciable hyperplastic effect on mouse epidermis, and were not further tested.

### METHODS.

*Mice.*—Stock albinos of the " S " strain (Salaman and Gwynn, 1951) were used. They were fed on rat cubes made according to the Rowett Institute formula (Thomson, 1930), and water *ad libitum.*

*Primary treatment.*—An initial application of 0·3 ml. of 0·1 or 0·15 per cent 9 : 10 dimethyl-1 : 2 benzanthracene (DMBA) in acetone was made to the whole back, after the hair had been clipped

*Interval.*—Before further treatment a period varying between 21 and 39 days was allowed to elapse. The exact length of this interval, in view of Berenblum's results with croton oil (Berenblum and Shubik, 1949*b*), was not considered to be critical.

*Secondary treatment.*—Substances to be tested for co-carcinogenic action were dissolved in acetone in concentrations found, in preliminary tests, to be the maximum tolerated without severe crusting or ulceration. Weekly, or occasionally twice a week, 0·3 ml. amounts of these solutions were applied to the previously treated areas.

In a few cases the concentration and frequency of these applications had to be varied during the course of the experiment, owing to the development of excessive crusting, as recorded below. Acetone (AR grade) was the solvent throughout, but in the case of Scarlet R, 10 per cent liquid paraffin, and in that of oestradiol, 10 per cent arachis oil, was added. These treatments were continued for periods varying from 140 to 200 days. They are recorded in Table II.

TABLE II.—*Incidence of Skin Tumours in Treated Mice.*

| Group. | No. of mice. | Primary treatment (DMBA %). | Interval in days. | Secondary treatment. Substance. | Duration. | Hyperplasia of epidermis. | Tumour bearing mice/survivors. | No. of tumours. |
|---|---|---|---|---|---|---|---|---|
| 1 | 12 | 0·15 | — | None | — | 0 | 2/9 | 2 |
| 2 | 16 | 0·1 | — | ,, | — | 0 | | |
| 3 | 12 | 0·15 | 21 | Acetone | Twice weekly, 12 weeks, weekly, 15 weeks | 0 | 1/12 | 1 |
| 4 | 13 | 0·15 | 21 | Croton oil 0·5% | As group 3 | ++ | 5/5 | 26 |
| 5 | 16 | 0·1 | 39 | ,, ,, 0·5% | Weekly, 20 weeks | ++ | 12/16 | 189 |
| 6 | 12 | 0·15 | 21 | Iodoacetic acid M/20–M/10 | As Group 3 | ++ | 8/10 | 54 |
| 7 | 12 | 0·15 | 21 | Chloroacetophenone M/40–M/20 | As Group 3 | ++ | 9/12 | 20 |
| 8 | 16 | 0·1 | 39 | Iodoacetamide M/30–M/15 | As Group 5 | ++ | 4/16 | 4 |
| 9 | 16 | 0·1 | 39 | Acetic acid M/10–M/5 | As Group 5 | 0 | 1/16 | 1 |
| 10 | 12 | 0·15 | 21 | Oestradiol 0·05% | As Group 3 | 0 | 0/6 | 0 |
| 11 | 12 | 0·15 | 21 | Scarlet R, saturated solution | As Group 3 | + | 2/10 | 3 |
| 12 | 12 | 0·15 | 21 | Oleic acid, undiluted | As Group 3 | + | 2/10 | 2 |
| 13 | 16 | 0·1 | 39 | Turpentine 50% | As Group 5 | ++ | 2/15 | 2 |
| 14 | 16 | 0·1 | 39 | Cantharadin 0·01–0·05% | As Group 5 | + | 0/5 | 0 |
| 15 | 16 | 0·1 | 39 | Podophyllin 0·1–0·3% | As Group 5 | ++ | 1/14 | 1 |
| 16 | 16 | 0·1 | 39 | Mustard oil 3·0–4·5% | As Group 5 | + | 1/16 | 2 |
| 17 | 19 | 0·15 | 21 | 1:4-naphthoquinone M/30 | Weekly, 27 weeks | ++ | 3/18 | 3 |
| 18 | 19 | 0·15 | 21 | 1:2-naphthoquinone M/30 | As Group 17 | 0 | 1/18 | 1 |
| 19 | 18 | 0·15 | 21 | 2 methyl-1:4-naphthoquinone M/30 | As Group 17 | ++ | 6/17 | 6 |
| 20 | 19 | 0·15 | 21 | Benzoqinone M/30 | As Group 17 | 0 | 0/13 | 0 |

*Biopsies.*—Pieces of skin, approximately 1 × 0·5 cm. in area, were removed under ether anaesthesia from the treated areas at intervals during the secondary treatments. Tissues were fixed in Zenkers fluid, and stained with haematoxylin and Eosin-Biebrich Scarlet [Eosin (water soluble) and Biebrich Scarlet each 0·5 g., glycerol 10 ml., methanol 40 ml., distilled water 450 ml.]

*Tumour incidence.*—Tumours were recorded weekly, and a final count made 3 days after the last treatment. They were observed visually for signs of malignancy, and sections were cut, after autopsy, in suspicious cases.

### RESULTS.

In general, 5–10 days after the primary painting with the carcinogen most mice showed some degree of epilation. About a third developed a mild degree of scabbing, or firm raised ridges or lumps of thickened skin, ususally in the scapular region, 10 to 15 × 2 to 3 mm. in area ; most of these were visible for only 2 to 3 weeks, though a few gave rise to tumours later. By the time secondary treatment was started the hair had grown, and there were only a small number of persistent thickenings and scars visible. Very little further epilation or crusting occurred during the secondary treatments, except in the cases of iodoacetic acid, chloroacetophenone, cantharidin, and podophyllin.

### *Tumour incidence.*

All tumours appeared to be benign papillomas macroscopically, of diameters 1 to 10 mm. No evidence of malignancy was found in the few suspicious tumours examined microscopically. Subsequent observation in this laboratory has shown (unpublished results) that a much longer time must elapse before an appreciable number of malignant tumours are seen after a small dose of a carcinogen followed by repeated treatment with a co-carcinogen.

A few tumours appeared in the control groups (1 and 2, no secondary treatment): 4 among 21 survivng mice ; and only one tumour among 12 surviving mice in Group 3 (acetone as secondary treatment).

In Groups 4 and 5 (croton oil as secondary treatment) there were 215 tumours on 17 surviving mice : many more were seen at an earlier date, but a number of mice bearing large numbers of tumours died before the end of the experiment.

Among the remaining groups, only in 6 and 7 (iodoacetic acid and chloroacetophenone as secondary treatments, respectively), was the tumour incidence significantly greater than that in the control group. The 10 surviving mice of Group 6 carried between them 54 tumours, while the 12 survivors of Group 7 carried 10 tumours between them, at the end of the experiment.

In Group 17 (1 : 4 naphthoquinone), 19 (2 methyl 1 : 4 naphthoquinone), 8 (iodoacetamide) and 11 (Scarlet R), the tumour incidence was slightly higher than that of the controls, but these results are not statistically significant.

Details of these results are found in Table II, and the course of tumour appearance for Groups 1, 4, 6, 7, and 19, expressed as the mean number of tumours per surviving mouse plotted against time, in Fig. 1.

By comparing these means at 15 and 22 weeks after the start of secondary treatments it was shown that tumour incidences in Groups 4, 6, and 7 are significantly higher than in Group 1 (controls,) and also different from each other, whereas the incidence in Group 19 is not significantly different from that of the

FIGURE 1.

● Group 1 (no secondary treatment).
+ Group 4 (secondary treatment : croton oil).
○ Group 6 ( „ „ : iodoacetic acid).
△ Group 7 ( „ „ : chloroacetophenone).
× Group 19 ( „ „ : 2-methyl 1:4-naphthoquinone).

All groups received one primary treatment with 9:10 dimethyl 1:2 benzanthracene 3 to 6 weeks before the beginning of secondary treatments.

controls, though the figures suggest that such a difference might have been observed in a larger experiment.

*Histology.*

All those substances which were definitely co-carcinogenic caused marked epidermal hyperplasia, but hyperplasia was also caused by substances that were not co-carcinogenic. A measure of the hyperplasia was obtained by counting the total number of cells per 1 mm. length of epidermis, excluding hair follicles.

In addition the percentage of " resting cells", i.e. basal cells proper (Glücksman 1945), in the epidermis of mice in 8 of the groups was determined by methods previously described (Salaman and Gwynn, 1951). A single count was made on the epidermis of mice taken from the selected groups, at about the time that tumours were beginning to appear in the croton oil-treated animals (59–66 days after the start of secondary treatment). Of the groups selected three had been treated with substances that produced both epidermal hyperplasia and tumours (croton oil, iodoacetic acid, and chloroacetophenone), and four with substances that caused epidermal hyperplasia but no tumours (iodoacetamide, oleic acid, Scarlet R, and mustard oil). The last substance caused only a weak hyperplasia.

The percentage of resting cells was significantly raised above that of the control group (1) in the chloroacetophenone and iodoacetic acid groups (6 and 7); as well as in the croton oil group (4) (as previously reported, Salaman and Gwynn, 1951), but not in any of the other groups.

The total number of cells per mm. of epidermis was significantly higher than in the control in all the selected groups, except that treated with mustard oil, and highest in the croton oil group. Total and resting cell counts are given in Table III.

### DISCUSSION.

Of the substances tested, iodoacetic acid and chloroacetophenone were found to possess marked co-carcinogenic power, though in the concentrations used this was inferior to that of croton oil. 2-Methyl-1 : 4 naphthoquinone slightly increased tumour incidence compared with controls ; this result is suggestive but, under the conditions of this experiment, it cannot be shown to be statistically significant. The effects produced by iodoacetamide, 1 : 4 naphthoquinone, and Scarlet R were even less definite. The other substances were inactive.

TABLE III.—*Differential Cell Counts in Treated Skin.*

| Group. | Secondary treatment. | Days after secondary treatment. | Resting cells as % of all epidermal cells. | Total cells per mm. length of epidermis. |
|---|---|---|---|---|
| 1 | None | 66 | 16·5±0·8 | 193± 5·5 |
| 4 | Croton oil | 66 | 34·2±0·8 | 355±23·9 |
| 6 | Iodoacetic acid | 66 | 23·6±0·7 | 258±12·6 |
| 7 | Chloroacetophenone | 66 | 22·5±0·6 | 282±11·3 |
| 8 | Iodoacetamide | 59 | 16·0±0·8 | 246±19·2 |
| 11 | Scarlet R | 66 | 16·2±0·8 | 250±16·7 |
| 12 | Oleic acid | 66 | 17·6±0·8 | 255± 8·4 |
| 16 | Mustard oil | 59 | 15·8±0·8 | 202± 5·8 |

All groups received one primary treatment with 9:10 dimethyl 1:2 benzanthracene 3 to 6 weeks before the beginning of secondary treatments.

From Table III it is evident that iodoacetic acid, chloroacetophenone, and croton oil (the co-carcinogenic substances) increase the percentage of resting cells in mouse epidermis previously treated with a carcinogen, while the non-co-carcinogenic substances tested do not. This suggests a connection between the two properties. The significance of such a connection is at present quite unknown.

Epidermal hyperplasia, however, measured as the increase in total number of cells per mm. length of epidermis, is not significantly greater in the co-carcinogenic than in the non-co-carcinogenic groups. Hyperplasia may be a necessary, but is not a sufficient, condition for co-carcinogenic action.

It is evident too, that the purgative or vesicant properties of the agents tested are not associated with co-carcinogenicity.

The occurence of two co-carcinogenic substances among the three –SH reactors tested is interesting. It has been suggested that carcinogens themselves may react with –SH groups (Crabtree, 1945, 1947; Calcutt, 1949). Crabtree (1940) found that a number of –SH reactors retarded tumour formation when applied alternatively with a carcinogenic hydrocarbon to mouse skin, but that one of them, chloracetone (Crabtree, 1941) slightly stimulated tumour formation when applied after a course of treatment with benzpyrene. The failure of iodoacetamide to produce tumours may have been due to faulty penetration or rapid removal. It is clear that so little is known of the effective concentration at susceptible sites in a tissue of applied substances of varying physical properties that it is not profitable to attempt to correlate observed co-carcinogenic potency with other chemical activity.

### SUMMARY.

1. A number of substances were tested for co-carcinogenic activity of the type shown by croton oil, namely the production of tumours when applied repeatedly to mouse skin after a single application of a carcinogenic hydrocarbon. They included substances which react with –SH groups, various quinones, oestradiol, and a group of miscellaneous skin irritants and purgatives.

2. Of these only two, iodoacetic acid and chloroacetophenone, both –SH reactors, gave rise to a significant tumour incidence.

3. Both these co-carcinogenic substances produced epidermal hyperplasia, but several of the non-co-carcinogenic substances had a similar effect.

4. Both iodoacetic acid and chloroacetophenone, when applied after the carcinogen as described, increased significantly the proportion of "resting cells" (Glücksmann, 1945) in the epidermis, as croton oil had been shown to do (Salaman and Gwynn, 1951). None of the other substances tested had this property.

We are indebted to D. I. Connell, W. J. Milton and J. Rawlings for skilled technical assistance.

The expenses of this research were partly defrayed out of a block grant from the British Empire Cancer Campaign.

### REFERENCES.

BERENBLUM, I.—(1935) *J. Path. Bact.*, **40**, 549. —(1944) *Arch. Path.*, **38**, 233. —(1951) *J. nat Cancer Inst.*, **11**, 839.

*Idem* AND SHUBIK, P.—(1947*a*) *Brit, J. Cancer*, **1**, 379. —(1947*b*) *Ibid*, **1**, 383. —(1949*a*) *Ibid*, **3**, 109. —(1949*b*) *Ibid*, **3**, 384.

BORST, M.—(1924) "Allgemeine Pathologie der Malignen Geschwülste", Leipzig (S. Hirzel).

BULLOUGH, W. S.—(1950) *J. Endocrinol.*, **6**, 340.

CALCUTT, G.—(1949) *Brit. J. Cancer*, **3**, 306.

CRABTREE, H. G.—(1940) *J. Path. Bact.*, **5**, 303.—(1941) *Cancer Res.*, **1**, 34.—(1945)*Ibid.*, **5**, 346.,—(1947) *Brit. med. Bull.*, **4**, 345.

DIXON, M., AND NEEDHAM, D. M.—(1946) *Nature*, **158**, 432.

FLAKS, J.—(1948) *Brit. J. Cancer*, **2**, 386.

*Idem* AND BER, A.—(1938*a*) *C. R. Soc. Biol., Paris*, **128**, 506.—(1938*b*) *Bull. Ass. franc. Cancer*, **28**, 108. —(1939) *Proc. 3rd int. Cancer Congress*, p. 160.
GLÜCKSMANN, A. (1945)—*Cancer Res.*, **5**, 385.
KING, L. S., AND CAULDWELL, E. W.—(1949) *J. nat. Cancer Inst.*, **10**, 131.
KLEIN, B. E., AND RUSCH, H. P.—(1944) *Cancer Res.*, **4**, 762.
ROUS, P., AND KIDD, J. G.—(1941) *J. exp. Med.*, **73**, 365.
SALAMAN, M. H.—(1952) *Brit. J. Cancer*, **6**, 155.
*Idem* AND GWYNN, R. H.—(1951) *Ibid*, **5**, 252.
SHUBIK, P.—(1950) *Cancer Res.*, **10**, 13.
TAKIZAWA, N.—(1940) *Proc. imp. Acad., Japan*, **16**, 309.
THOMSON, W.—(1930) *J. Hyg.*, **36**, 24, 156.
TWORT, J. M., AND TWORT, C. C.—(1939) *Amer. J. Cancer*, **35**, 80.
VISSER, J., AND TEN SELDAM, R. E. J.—(1938) *Geneesk. Tidjschr. Ned.-Ind.*, **78**, 3280.